# The Topological Properties of Stimuli Influence Fear Generalization and Extinction in Humans

**DOI:** 10.3389/fpsyg.2018.00409

**Published:** 2018-03-28

**Authors:** Liang Xu, Hongyu Su, Xiaoyuan Xie, Pei Yan, Junjiao Li, Xifu Zheng

**Affiliations:** ^1^School of Psychology, South China Normal University, Guangzhou, China; ^2^Department of Humanities and Social Sciences, Guangdong Communication Polytechnic, Guangzhou, China; ^3^Center for Studies of Psychological Application, South China Normal University, Guangzhou, China; ^4^Guangdong Key Laboratory of Mental Health and Cognitive Science, South China Normal University, Guangzhou, China

**Keywords:** fear generalization, topological property, generalization stimulus, fear extinction, return of fear

## Abstract

Fear generalization is an etiologically significant indicator of anxiety disorders, and understanding how to inhibit it is important in their treatment. Prior studies have found that reducing fear generalization using a generalization stimulus (GS) is ineffective in removing a conditioned fear that incorporates local features, and that topological properties appear to play a comparatively more significant role in the processes of perception and categorization. Our study utilized a conditioned-fear generalization design to examine whether the topological properties of stimuli influence the generalization and return of fear. Fear was indexed using online expectancy ratings and skin conductance responses (SCRs). The study’s 52 participants were divided into three groups: Group 1, conditioned danger cue (CS+) extinction; Group 2, extinction of one GS; Group 3, extinction of three GSs. We found that the three groups acquired conditioned fear at the same level. In the generalization and extinction phase, fear was transferred to the GS with the same topological properties as CS+, and gradual decreases in both shock expectancy and SCRs over non-reinforced extinction trials were observed. In the test phase, participants’ online expectancy ratings indicated that fear did not return in Group 1, but did return in Groups 2 and 3. All three groups demonstrated successful GS fear extinction, but only Group 1 did not show a return of fear for CS+. Regarding SCRs results, none of the groups demonstrated a return of fear, suggesting that utilization of topological properties successfully reduced the return of conditioned fear. Our results indicate that, in clinical settings, using GS with topological equivalence to CS+ might offer a potential method with which to extinct conditioned fear.

## Introduction

An originally neutral stimulus can acquire predictive properties as a conditioned stimulus (hereafter, “CS”), and thereby elicit conditioned emotional behaviors and responses (a “conditioned response”), if it is repeatedly paired with an unconditioned stimulus (“US”) (Pavlov, 1927). Conditioned responses can also generalize to stimuli that resemble the original CS (generalization stimulus; hereafter, “GS”). The study of fear generalization processes such as these has historically been undertaken using animals (Pavlov, 1927), but the research has recently been extended to include human subjects (Vervliet et al., 2004, 2005; Lissek et al., 2008, 2010; Dunsmoor et al., 2009). In investigations of the perceptual generalization of fear in humans, visual conditioned and generalization stimuli comprising rings of gradually increasing size are commonly used (Lissek et al., 2008, 2010, 2014; Lissek and Grillon, 2012; Xu et al., 2016), wherein two extreme ring sizes serve as the conditioned danger cue (CS+) and the conditioned safety cue (CS-), respectively, with the CS+ and CS- counterbalanced over the smallest and largest ring. Alternatively, visual stimuli of a particular color and shape have been used as the CS and GS (Vervliet et al., 2010; Vervliet and Geens, 2014; Ahmed and Lovibond, 2015). However, studies that use such stimuli only focus on a single physical characteristic, and therefore do not accurately reflect how we perceive our natural environment. In reality, when we perceive fearful objects or experience traumatic events, we will generalize the conditioned fear to a new object or context based on regularities that go beyond physical resemblance (Dymond et al., 2015); that is, we tend to process the stimulus as a whole or as an entire concept (Pomerantz et al., 1997; Chen, 2005). Furthermore, visual perception is sensitive to the topological properties of stimuli. In topological property, a difference in a figure’s size is not considered, but properties such as whether the stimulus is closed or open, bound or unbound, or simply connected or multiply connected are considered. Among the most commonly perceived topological properties are the connectivity of the stimulus and the number of holes it has (Chen, 1990).

In typical perceptual generalization research, GSs are interchangeable—for example, eight rings of gradually increasing size (Lissek et al., 2008). However, a key step concerning perception is the selection of which aspect of a stimulus will be used as a category standard (Mai and Lei, 2000). A variety of studies suggests that the topological properties of a stimulus play fundamental roles in the processes of perception and categorization (Chen, 1982, 1990). The topological features ensure that the GSs belonging to a category are interchangeable. In addition, given that the topological features result in two different categories, it seems fair to assume that not much (if any) generalization should be expected between the CS- and the CS+ and that a specific GS will only receive generalization from the CS that defines the category (i.e., a GS+ receives mainly generalization from the CS+ and not from the CS-, while a GS- receives mainly generalization from the CS- and not the CS+).

Topological equivalence is mainly reflected during shape changes; that is, when a stimulus smoothly changes its shape without breaking or fusing, the topological properties remain unchanged as long as the connectivity and number of holes remain the same (Mai and Lei, 2000; Zhuo et al., 2003). For example, when a triangle turns into a square or circle, because the connectivity is invariant, the topological properties remain unchanged. Similarly, a doughnut and coffee cup are considered topologically equivalent since they both have a hole in the center. Topological theory proposes that perception is based on the transformative and the invariant properties of stimuli (Chen, 1982, 1990). With regard to topological perception, compared to the processing of local geometric properties and physical characteristics of a stimulus, the processing of topological properties occurs in the early stage of visual perception (Chen, 1982). Moreover, topological properties may be regarded as an informative cue to guide attention to the target more efficiently. Specifically, topological equivalence has a significant influence in maintaining the contextual cuing effect (Ma et al., 2018). For example, if a person is bitten by a dog, the fear will be generalized to other dogs, based on topological properties or categories rather than on local properties, such as its size, shape, or the color of its hair. Moreover, if the stimuli are temporary, after the stimuli disappear, the affected person will use their short-term memory to code them. In such instances, the most important information in the perception that comprised topological properties would determine the results of coding, and thus influence perceptual categorization (An et al., 2004).

In clinical settings, patients exhibiting excessive fear generalization might have certain anxiety disorders, and such fear generalization may potentially be an etiologically significant indicator of anxiety disorders (Lissek et al., 2008). Overgeneralization of conditioned fear has been documented in panic disorder, post-traumatic stress disorder and generalized anxiety disorder (Lissek et al., 2010, 2014). Thus, establishing how to effectively inhibit this overgeneralization of fear may be key to treating such anxiety disorders. Although a former study showed that extinction of a CS+ could effectively reduce the generalization level of fear (Vervliet and Geens, 2014), in actual clinical therapy, it is usually not possible to use the original CS+ to counteract fear, especially if it is abstract, ethically forbidden or related to a physical condition. Hence, almost all of the stimuli that are used clinically to extinguish conditioned fear are GSs. However, Vervliet et al. (2004) also found that the extinction using a GS could not bring about the elimination of a conditioned fear incorporating local features. Because topological features play an important role in perception and categorization, in our study, we utilized topological features but not local features, such as the shape or the size, to investigate whether a GS with the same topological features as the CS+ could influence its fear generalization and extinction.

In the present study, we used the topological property that best fitted the perceptual process in order to assess the influence of topological equivalence on the generalization and extinction of fear. Moreover, we used GSs that were topologically, but not physically, equivalent to the CS+ in order to determine whether the elimination of the GS can effectively influence the level of fear elicited by the CS+. Based on existing evidence (An et al., 2004; Vervliet and Geens, 2014), we hypothesized that the topological equivalence of the stimuli would influence both the generalization and the extinction of fear. Because the GSs were not interchangeable and of topological equivalence with the CS, the generalization of fear could confirm the topological features play important roles in the processes of perception and categorization. Further, if fear could be successfully removed by using stimuli of topological equivalence to the CS+, this could point toward a new approach in treating conditioned fear and anxiety disorders.

## Materials and Methods

### Participants

All the experimental procedures within this study were approved by the ethics committee of South China Normal University. All participants provided written informed consent prior to participating in the study, and they were modestly compensated (20 Yuan RMB) for their participation.

Fifty-six undergraduate students from South China Normal University participated in this study. All participants were right-handed, with normal or corrected-to-normal vision. Participants were randomly assigned to one of three groups: Group 1, CS+ extinction; Group 2, extinction of one GS; and Group 3, extinction of three GSs. Four participants were excluded from the final analysis owing to technical problems and voluntary withdrawal; thus, the data from 52 participants were analyzed. There were no significant differences noted in respect of age and anxiety scores, the latter of which were measured using the State-Trait Anxiety Inventory (STAI) (Spielberger et al., 1970; Chinese version, Wang et al., 1999). Descriptions of the study’s participants are given in **Table 1**.

**Table 1:** Descriptions of the participant groups.

Variable	Group 1^a^		Group 2^b^		Group 3^c^		Significance
	Mean	*SD*	Mean	*SD*	Mean	*SD*	
Age (year)	19.33	1.19	19.35	1.06	19.24	1.09	*p* > 0.05
STAI–State	35.06	3.30	33.59	1.87	33.35	2.15	*p* > 0.05
STAI–Trait	36.06	2.84	36.53	2.43	35.24	3.33	*p* > 0.05

^a^*n = 18 (10 female). ^*b*^*n* = 17 (9 female). ^*c*^*n* = 17 (8 female)*.

### Apparatus

**FIGURE 1:**
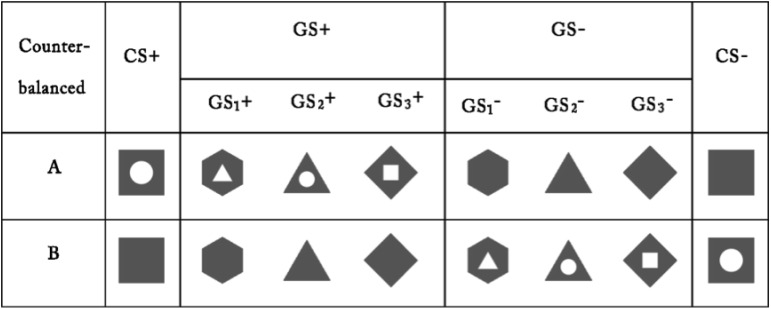
Conditioning and generalization stimuli. The chart depicts the conditioned danger stimulus (CS+), the conditioned safety stimulus (CS–) and the generalization stimuli (GS) used in the experiment.

The US was an electric shock delivered to the left wrist (using a Grass SD9 Square Pulse Stimulator; West Warwick, RI, United States), which was deemed individually by participants to be “highly uncomfortable but not painful” (Lissek et al., 2008). In the warm-up procedure, after a stimulating bar electrode had been attached to their left wrist and a gel applied between the skin and electrode (Li et al., 2017), each participant sat in front of a computer monitor and was asked to rate the degree of pain experienced through the electric shocks that were subsequently delivered. Participants rated the unpleasantness of each shock using a 9-point scale, which ranged from “1” = *no feeling of pain* to “9” = *painful*, by pressing the corresponding number key with their right hand. The question “How painful was the electrical stimulus?” was presented at the top of the screen and the 9-point scale at the bottom. The electric shock intensity started at 1 mA and was gradually increased by 0.2 mA increments. Individual warm-up procedures were concluded as soon as the participant pressed the “8” key, which denoted “*highly uncomfortable but not painful*.” Participants were then asked to confirm that the most intense of the electric shocks experienced was the final one that had been delivered, and consent was obtained for their participation in the remainder of the experiment. For the CS and the GS, we employed different images of geometric figures. For half of the participants, a gray square with a white circle in the middle and a completely gray square served as the CS+ and CS-, respectively; additionally, three gray geometric figures that had the same topological property as the CS+ (i.e., a white area in the middle) were used as the GS+, and three gray geometric figures that had the same topological property as the CS- (i.e., a completely gray triangle) were used as the GS-. For the other half, the stimuli were reversed. The color of the outline for the CS and the GS was black, and the size and resolution of the images were identical (see **Figure 1**).

Participants’ expectation of a shock being delivered was measured online during the presentation of each stimulus. The question “Is there an electric shock?” was presented above the CS/GS on the screen, and a 9-point scale, ranging from “1” = *certainly no electric shock* to “9” = *certainly an electric shock*, was presented below the CS/GS. Participants were prompted to rate their expectancy level of receiving the US by pressing the corresponding number key. After participants had pressed the key, the question “Is there an electric shock?” and the 9-point scale, along with the CS/GS, would remain visible for a further 500 ms. The electric shock would be delivered during the 500 ms period before some CS+ offsets, and the CS and US were co-terminated.

A Spirit NeXus-10 Bio Trace system (BioTrace Medical, San Carlos, CA, United States) was used to measure participants’ skin conductance responses (SCRs). Two Ag/AgCl electrodes of a preformed size (20 × 16 mm) were attached to the tips of the second and third fingers of each participant’s left hand using adhesive tape. The electrodes were connected to the GSR100c module, which recorded the SCRs at 120 Hz. SCR waveforms were analyzed offline using BioTrace+ software for NeXus-10 (Li et al., 2017). SCRs elicited by the CS/GS were determined by calculating the difference between the peak response during 3–6 s after stimulus onset and a baseline average (i.e., 5 s before stimulus onset) in every single trial. A minimum response criterion of 0.02 microsiemens (μS) was used (Wolfram et al., 2012). All other responses were scored as zero and remained in the analyses (Effting and Kindt, 2007; Kindt and Soeter, 2013; Zeng et al., 2014). The raw SCR scores were square-root transformed to normalize the distribution (Schultz et al., 2013).

### Procedure

Prior to the start of the experiment, all study procedures were explained in detail to the participants, and any questions they had were answered. Once the SCR electrodes were attached, the participants were informed that they were about to see geometrical figures presented on the screen and that some of these presentations would be followed by an electric shock (of the same intensity as the final one that had been delivered to them in the warm-up procedure), while others would not. Participants were asked to rate their expectancy of receiving the US after the presentation of a figure by pressing the corresponding number key with the right hand, as described above, when the slide was presented (prior to the US). At the beginning of each trial, participants focused on a cross for 500 ms, and then the CS or GS was presented for 8 s. The question “Is there an electric shock?” and the 9-point scale were presented immediately after CS/GS offset. The electric shock was delivered 500 ms before CS+ offset. The inter-trial interval varied between 13 and 17 s, with a mean of 15 s (Schultz et al., 2013; Vervliet and Geens, 2014; Du et al., 2015).

The experiment consisted of three phases, as follows:

(1) *acquisition*—the CS+ and CS- were presented six times each (with a reinforcement ratio of 67%, which were four of the six CS+ co-terminated with shock delivery), in a randomized order and with the restriction that no more than two identical stimuli could be presented consecutively;
(2) *generalization and extinction*—the CS+ and CS- were presented six times each in Group 1, while, in Group 2, the GS+ (one of the three GS+ was selected at random) and GS- (one of the three GS- was selected at random) were presented six times each. In Group 3, all three GS+ and GS- were presented two times each. None of the CSs or GSs was paired with the US. The CS and GS were presented in a randomized order, with the restriction that no more than two identical stimuli could be presented consecutively; and
(3) *test*—eight stimuli were each presented once without a shock.

The presentation of the first stimulus was counterbalanced across participants and groups. The rest of the stimuli were presented in random sequences.

To perform the experiment, participants sat at a table facing a 21 inch liquid crystal display monitor in a sound-attenuated and air-conditioned room (25°C). The software package E-Prime 2.0 was used for stimuli presentation and data collection.

### Data Analysis

SPSS 25.0 was used to analyze the data. A trial (6) × stimulus type (2) × group (3) repeated measures analyses of variance (RM-ANOVA) was used to analyze the acquisition phase data. The generalization and extinction phase data were analyzed with a trial (6) × stimulus type (2) × group (3) RM-ANOVA, multiple *post hoc* tests, separate paired-samples *t*-tests and a one-way ANOVA. Finally, a stimulus type (8) × group (3) RM-ANOVA and a one-way ANOVA were used to analyze the test phase data.

Differences were considered statistically significant at *p* < 0.05. *Post hoc* tests were performed using Fisher’s least significant difference test between groups. We adopted $\eta_{p}^{2}$ as the estimate of partial effect size and used Greenhouse–Geisser corrections for violations of sphericity when appropriate.

## Results

### Online Expectancy Ratings

#### Acquisition Phase

A trial (6) × stimulus type (2) × group (3) RM-ANOVA of the US expectancy ratings for the acquisition phase did not reveal a significant main effect of group [*F*_(2,49)_ = 0.75, *p* > 0.05, $\eta_{p}^{2}$ = 0.01]. The main effect of stimulus type was significant [*F*_(1,49)_ = 373.54, *p* < 0.001, $\eta_{p}^{2}$ = 0.88], as was the main effect for trial [*F*(_5,245)_ = 2.77, *p* < 0.05, $\eta_{p}^{2}$ = 0.05]. Additionally, a significant interaction effect was identified between the stimulus type and trial [*F*_(5,245)_ = 135.46, *p* < 0.001, $\eta_{p}^{2}$ = 0.73]. *Post hoc* analysis showed, from the third trial, the ratings for the CS+ began to be significantly higher than for the CS- (*p* < 0.001). The data indicated that, during this phase, participants learned the CS–US combination and that the three groups acquired conditioned fear at the same level.

#### Generalization and Extinction Phase

A trial (6) × stimulus type (2) × group (3) RM-ANOVA of the US expectancy ratings revealed significant main effects for trial, stimulus type and group [trial: *F*_(5,245)_ = 145.14, *p* < 0.001, $\eta_{p}^{2}$ = 0.75; stimulus type: *F*_(1,49)_ = 395.56, *p* < 0.001, $\eta_{p}^{2}$ = 0.89; group: *F*_(2,49)_ = 17.04, *p* < 0.001, $\eta_{p}^{2}$ = 0.41]. In addition, significant stimulus type × group, stimulus type × trial, group × trial and stimulus type × trial × group interaction effects emerged (all *F* > 3.23, *p* < 0.001, $\eta_{p}^{2}$ *>* 0.12). *Post hoc* testing revealed that Group 1 was significantly different from Groups 2 and 3 (both *p* < 0.001), while no difference was identified between Groups 2 and 3 (*p* > 0.05). The US expectancy ratings for CS+ in Group 1 remained until the sixth trial, while the ratings for GS+ in Groups 2 and 3 began to extinct from the fourth trial. In all three groups, the ratings on CS+/GS+ were significantly higher than those for CS-/GS- (all *p* < 0.001).

Collectively, the above results suggest that a topological property could produce fear generalization and gradual decreases in shock expectancy over non-reinforced extinction trials. Compared with GS+ in Groups 2 and 3, the US expectancy ratings for CS+ in Group 1 were more enduring.

**FIGURE 2:**
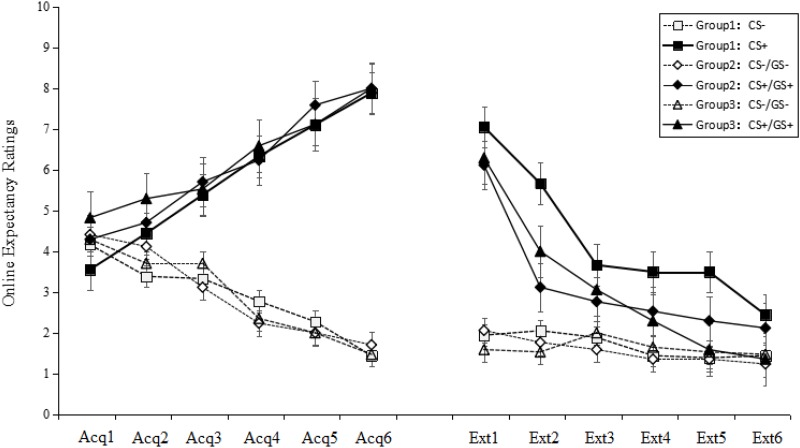
Mean expectancy ratings during the acquisition phase and the generalization and extinction phase. On the *x*-axis, “Acq1” to “Acq6” refer to the ratings for the CS+ and CS– during the six acquisition trials, which indicate that participants learned the CS–US contingency and all three groups acquired conditioned fear at the same level. “Ext1” to “Ext6” refer to the ratings for the CS+/GS+ and CS–/GS– during the generalization and extinction phase, which indicate that Groups 2 and 3 that were topologically equivalent with CS+ (Group 1) could produce fear generalization, and the fear gradually decreased over the non-reinforced extinction trials. Error bars represent standard errors of mean.

There were no significant differences among the six figures taken as GS+ [*F*_(5,33)_ = 1.40, *p* > 0.05, $\eta_{p}^{2}$ = 0.01] in the first trial concerning generalization. This result implies that the six GSs were equivalent in the generalization (see **Figure 2**).

#### Test Phase

A stimulus type (8) × group (3) RM-ANOVA of the US expectancy ratings during the test phase revealed a significant main effect of stimulus type [*F*_(7,343)_ = 79.36, *p* < 0.001, $\eta_{p}^{2}$ = 0.62], a significant main effect of group [*F*_(2,49)_ = 16.66, *p* < 0.001, $\eta_{p}^{2}$ = 0.41] and a significant stimulus type × group interaction effect [*F*_(14,343)_ = 20.70, *p* < 0.001, $\eta_{p}^{2}$ = 0.46]. *Post hoc* testing revealed that the ratings in Group 1 were significantly higher than those of Groups 2 and 3 (both *p* < 0.001), while no difference was identified between Groups 2 and 3 (*p* > 0.05). In Group 1, there were no significant differences among the stimulus types (all *p* > 0.05). However, in both Groups 2 and 3, CS+ were significant higher than CS- (all *p* < 0.001), and there were no significant differences between GS_1_+, GS_2_+, GS_3_+ and CS- (all *p* > 0.05). Specifically, the ratings for the CS+ in Groups 2 and 3 were not significantly different, but the ratings in both groups were higher than the ratings in Group 1.

**FIGURE 3:**
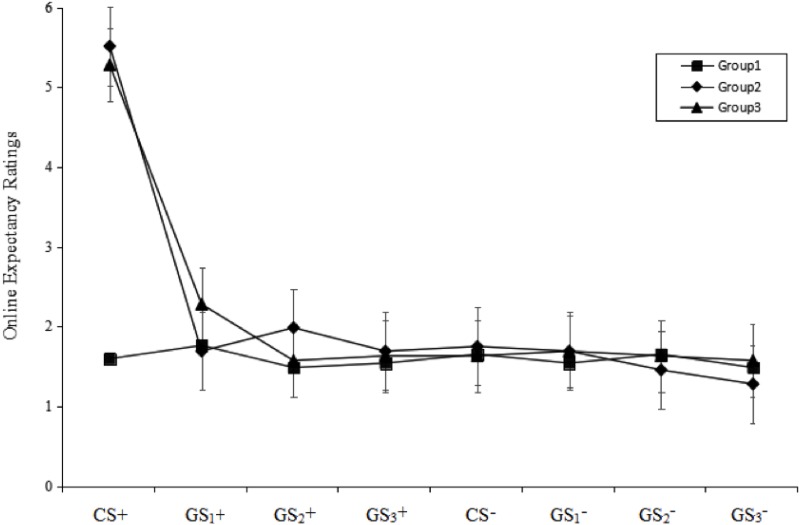
Mean expectancy ratings during the test phase. The figure indicates that only Group 1 did not show a return of fear for the CS+ (i.e., neither Group 2 nor Group 3 eliminated the fear of CS+). All three groups successfully eliminated the fear of GS+. Error bars represent standard errors of mean.

These results indicate that fear extinction could be transferred from the CS+ to the GS+, but not from the GS to the CS+. Additionally, extinction of the GS+ could be transferred to other GS+ that share the same topological property (see **Figure 3**).

### Skin Conductance Responses

#### Acquisition Phase

A trial (6) × stimulus type (2) × group (3) RM-ANOVA of the SCRs in the acquisition phase did not reveal a significant effect of group [*F*_(2,49)_ = 1.19, *p* > 0.05, $\eta_{p}^{2}$ = 0.008]. However, there was a significant main effect of stimulus type [*F*_(1,49)_ = 253.85, *p* < 0.001, $\eta_{p}^{2}$ = 0.84] and a significant main effect of trial [*F*_(5,245)_ = 21.54, *p* < 0.001, $\eta_{p}^{2}$ = 0.31]. Moreover, a significant stimulus type × trial interaction effect was found [*F*_(5,245)_ = 84.73, *p* < 0.001, $\eta_{p}^{2}$ = 0.63]. *Post hoc* analysis showed, from the third trial, a significance of SCRs for the CS+ rather than for the CS- (*p* < 0.001), which was characterized by higher SCRs for the CS+ than for the CS-. The data also indicated that all participants acquired conditioned fear at the same level.

#### Generalization and Extinction Phase

A trial (6) × stimulus type (2) × group (3) RM-ANOVA of the SCRs revealed significant main effects for trial, stimulus type and group [trial: *F*_(5,245)_ = 127.99, *p* < 0.001, $\eta_{p}^{2}$ = 0.72; stimulus type: *F*_(1,49)_ = 446.20, *p* < 0.001, $\eta_{p}^{2}$ = 0.90; group: *F*_(2,49)_ = 4.13, *p* < 0.05, $\eta_{p}^{2}$ = 0.14]. No significant interaction effects were found (all *F* < 1.59, *p* > 0.05, $\eta_{p}^{2}$ < 0.09). *Post hoc* testing revealed that the SCRs in Group 1 were significantly higher than in Groups 2 and 3 (both *p* < 0.001), while no difference was identified between Groups 2 and 3 (*p* > 0.05). In all three groups, the SCRs on CS+/GS+ were significant higher than those for CS-/GS-, indicating that topological properties can produce fear generalization, and the SCRs gradually decreased over the course of the experiment.

**FIGURE 4:**
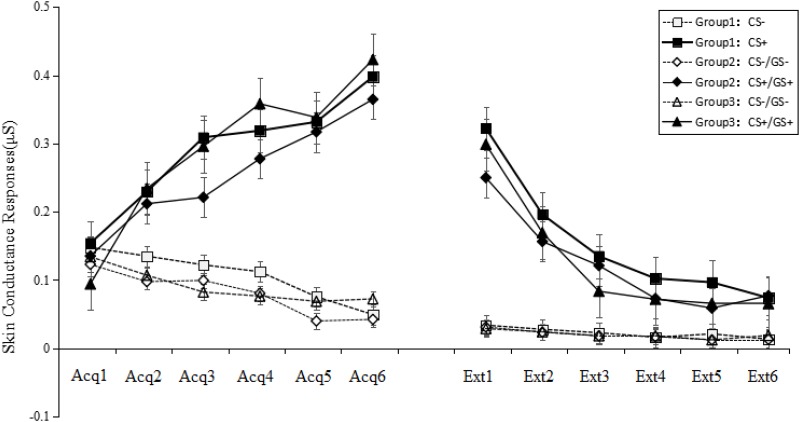
Mean SCRs during the acquisition phase, and generalization and extinction phase. On the *x*-axis, “Acq1” to “Acq6” refer to the SCRs for the CS+ and CS– during the six acquisition trials, which indicate that all participants acquired conditioned fear at the same level. “Ext1” to “Ext6” refer to the SCRs for the CS+/GS+ and CS–/GS– during the generalization and extinction phase, which indicate that Groups 2 and 3 that were topologically equivalent with CS+ (Group 1) could produce fear generalization, and the SCRs gradually decreased over the course of the phase. Error bars represent standard errors of mean.

There were no significant differences among six figures taken as GS+ [*F*_(5,33)_ = 0.60, *p* > 0.05, $\eta_{p}^{2}$ = 0.007] in the first trial concerning generalization. This result implies that the six GSs were equivalent in the generalization (see **Figure 4**).

#### Test Phase

**FIGURE 5:**
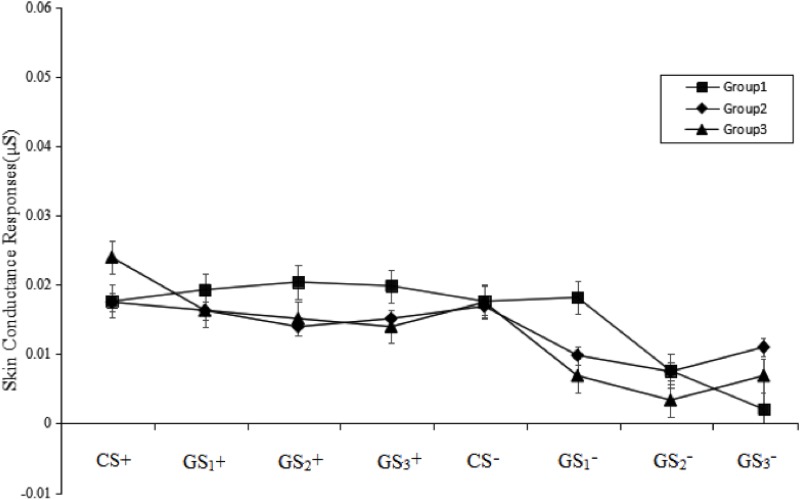
Mean SCRs during the test phase. The figure indicates that none of the groups demonstrated a return of fear for the CS+ and GS+. Error bars represent standard errors of mean.

A stimulus type (8) × group (3) RM-ANOVA of the SCRs during the test phase revealed the main effect of group was not significant [*F*_(2,49)_ = 1.06, *p* > 0.05, $\eta_{p}^{2}$ = 0.02]. However, a significant main effect of stimulus type [*F*_(7,343)_ = 11.55, *p* < 0.001, $\eta_{p}^{2}$ = 0.19] and a significant stimulus type × group interaction effect [*F*_(14,343)_ = 1.80, *p* < 0.05, $\eta_{p}^{2}$ = 0.07] were observed. *Post hoc* testing revealed that, regarding GS_1_-, Group 1 was significantly higher than Group 2 (*p* < 0.05) and Group 3 (*p* < 0.001). Regarding GS_3_-, Group 2 was significantly higher than Group 1 (*p* < 0.001). Regarding CS+, GS_1_+, GS_2_+, GS_3_+, CS-, and GS_2_-, there were no group differences. There were no significant differences between CS+, GS_1_+, GS_2_+, GS_3_+, and CS- in all three groups (all *p* > 0.05). These results suggest that the CS+, GS_1_+, GS_2_+, and GS_3_+ eliminated fear in the SCRs and that the extinction levels were not significantly different among the three groups (see **Figure 5**).

## Discussion

In the present study, by employing a basic conditioned-fear generalization paradigm, geometrical figures with the same topological property as the CS+ were used as a GS to study the influence of topological equivalence on the generalization and extinction of conditioned fear. We found that fear was successfully generalized from the CS+ to the GS with the same topological properties. Furthermore, the shared topological property successfully reduced the return of fear in the SCRs, but not in the online expectancy ratings.

During perception, the topological properties of objects (based on, e.g., physical connectivity or number of holes) are processed prior to the local geometric properties (Chen, 2005). In our study, the GS had a white area in the middle that was equivalent to that of the CS+, and this topological equivalence effectively induced fear generalization. The only difference between the CS+ and CS- was the presence of a white circle in the middle; hence, “a white circle” was the fear-relevant feature. This finding is in line with configural theories of associative learning, which assume that stimuli acquire associations as a whole, but the generalization is determined by the proportion of unique versus common features (Pearce, 1987). In differential CS+/CS- conditioning, the CS+ acquires excitatory strength (fear-eliciting properties) and the CS- acquires inhibitory strength (safety-eliciting properties) (Vervliet and Geens, 2014; Zeng et al., 2014). Depending on the similarity of the CS+/CS-, the CS+ receives generalized inhibitory strength from the CS- and excitation strength from the CS+. Their relative strengths determine the size of the conditioned response (larger in relation to the CS+ than to the CS-). It follows that the CS+ is not a simple fear elicitor, but instead involves complex interplay between its fear- and safety-eliciting properties. Based on this theory, GSs that resemble the CS+ more than they do the CS- will receive more generalized excitation than generalized inhibition, and will thus produce a generalized fear reaction (Vervliet and Geens, 2014). In the present study, if participants remembered the local features—take counterbalancing A for example—the participants might remember a gray square and a white circle in the middle. In the generalization phase, only GS_2_+ had the same local feature as CS+, in having a white circle, while GS_1_+ and GS_3_+ did not have the same local feature as CS+. However, GS_1_+, GS_2_+, and GS_3_+ did have the same topological features as CS+. If the local feature played a more important role in generalization, GS_2_+ might have demonstrated more generalization. However, GS_1_+, GS_2_+, and GS_3_+ showed the same generalization tendency.

The results we obtained in the fear acquisition phase suggest that the participants might have remembered the topological properties of the dangerous stimuli rather than the local features. On the other hand, CS+ (again, taking counterbalancing A as an example) can also be deemed as a compound stimulus, which consisted of a gray square and a white circle. In a prior study, compound stimuli were used (e.g., blue triangle, yellow square) to study which local features (such as shapes or colors) are more important in fear generalization, and it was found that verbal instruction focusing on one feature could transmit information about the relative importance of an individual stimulus features in terms of fear generalization (Vervliet et al., 2010; Ahmed and Lovibond, 2015). However, in the present study, GS_1_+ and GS_3_+ did not have any of the same local features (e.g., a gray square and a white circle) as CS+, but shared topological properties, which further verifies the importance of topological properties with respect to fear generalization. This finding is supported by previous studies showing that topological properties are superior to local features in the process of perception (Chen, 1982, 2005; Chen and Zhou, 1997; Huang et al., 2011). Indeed, this preeminence has been demonstrated both in cognitive neuroscience studies, which have found that the human brain, particularly the left temporal area, is sensitive to the topological properties of objects (Wang et al., 2007; Zhou et al., 2008; Zhuo et al., 2003), and in phylogenetic studies, which have revealed that this topological preference exists in the visual systems of lower animals too (Chen et al., 2003; Zhu et al., 2010). Together with these previous studies, our results indicate that topological properties play an important role not only during perception but also in fear generalization.

In our study, the extinction of the fear responses to the CS+ and GS was successful in all three groups according to the SCR measures during the test phase. However, in the online expectancy ratings, only Group 1, which had eliminated the fear responses to the CS+, successfully extinguished the fear responses to both the CS+ and GS. Conversely, Groups 2 and 3, which had to extinguish fear responses to one and three GSs, respectively, still had higher fear responses to the CS+. These findings are consistent with earlier studies on perceptual generalization and the extinction of fear that posit that eradicating a different-but-similar stimulus has little effect on the fear extinction of the original CS+ (Dubin and Levis, 1975; Vervliet et al., 2004, 2005, 2006, 2010; Boddez et al., 2012; Vervliet and Geens, 2014).

In addition, the lack of a significant difference between Groups 2 and 3 indicates that the number of GSs may not influence the return of fear. In Group 2, the extinction effect of the single GS transferred to the other GS during the test phase. As noted in the introduction, topological properties play a key role in the categorization of objects (Mai and Lei, 2000). In this study, all three GSs were topologically equivalent; hence, participants might have placed them in the same category. In the real world, complex objects and situations can be represented across a variety of dimensions, with any associated fear generalization spread over arbitrary stimuli with little perceptual overlap except for category (Vervoort et al., 2014). One approach to comprehending the complexity of human fear generalization incorporates theoretical knowledge, including categorization, and the organization of conceptual knowledge (Dunsmoor and Paz, 2015). Humans can use category-level knowledge to associate the GS with an entire category (Dunsmoor and Murphy, 2015). Thus, according to conceptual generalization, the extinction of fear for one object might reduce the fear for other objects in the same category (Vervliet et al., 2010; Vervoort et al., 2014). As for the present study’s CS+, although it belonged to the same category as the GS, its presence during fear acquisition established it as a cue of fear, and therefore it was considered to be different from the other stimuli. Similarly, in real life, a stimulus with intense fear relevance, such as a fearful face, would capture our attention more automatically than would a neutral face, and so the fearful face would be categorized differently (Öhman and Mineka, 2001; Öhman et al., 2001).

Here, we found a dissociation between the expectancy ratings and the SCRs, which persisted throughout the test phase. In the online expectancy ratings, Groups 2 and 3 both demonstrated the return of fear and had higher fear ratings than did Group 1. However, the SCR data did not show a return of fear or differences among the groups. The dissociation effects observed in the present study are consistent with those reported previously (Walther et al., 2009; Balderston and Helmstetter, 2010; Klucken et al., 2013; Vervoort et al., 2014), and these findings collectively support the idea that different types of memories are formed during the same training procedure. From the perspective of dual process theory, implicit and explicit performances are dissociable from one another (Knight et al., 2003; Vervoort et al., 2014; Feng et al., 2017). Additionally, they are concerned with two distinct memory systems: the former involves a declarative memory of the learned fear association between the CS and US, while the latter involves a procedural memory pertaining to the acquisition and expression of a fear response (Knight et al., 2003; Balderston and Helmstetter, 2010; Schultz et al., 2013). The results of the present study show that, even though the participants did not feel fear at an implicit physiological level, they tended to report higher fear expectancy, which is consistent with the results of former studies (Lissek et al., 2008; Haddad et al., 2013; Xu et al., 2016). In addition, SCRs not being inclined toward a stronger generalization of fear elimination from GSs to the CS+ might also indicate the absence of the return of fear on the physiological level, compared to the expectancy ratings (Zeng et al., 2014; Xu et al., 2016; Feng et al., 2017). Nevertheless, based on our findings and former studies, we posit that the larger generalization in the expectancy ratings than in the SCRs may be a robust phenomenon. In the explicit performances, some details of the stimuli may have been ignored, with participants therefore inclined to report a higher degree of fear. It is also possible that the reports of higher fear expectancy were related to participants’ attending to and collecting information about the threat stimulus (LeDoux, 2014). From an evolutionary point of view, increased reporting of fear could help us attract the attention of others to warn them of potential danger and/or obtain help (Xu et al., 2016).

One limitation of the present study was that, based on the expectancy ratings, the GS appeared to only receive a partial generalization of fear, which in turn likely produced less extinction learning. Less extinction learning implies less extinction generalization (Vervliet et al., 2004). Since our participants did not consider the GS equally as dangerous as the original CS+, the difference in extinction generalization may be just a difference in extinction learning.

Despite the above limitation, the present study successfully highlights the importance of topological properties in the processes of human fear generalization and extinction. As enhancing perceptual processing shows promise as an effective treatment for targeting excessive fear generalization (Struyf et al., 2017), topological properties could play a key role in perceptual processing and in connecting perceptual generalization with conceptual generalization. In exposure therapy, clinicians tend to use GS since the original CS+ often cannot be used during treatment. Therefore, understanding which GS will be the most effective is essential. The findings of the present study suggest that a GS with topological equivalence to the CS+ might be better than a GS with similar local features to the CS+. However, topological properties were shown to eliminate the fear for CS+ only in SCRs; the matter of how to use topological properties to effectively decrease fear generalization in clinical contexts needs further investigation. In reality, to face the same threatening stimuli or circumstance is rare. In the study, most stimuli of fear were GS similar to the original one, and topological equivalence with the CS+ was superior. These results suggest that fear extinction could transfer to other stimuli sharing the same topological properties, affirming that topological properties might be beneficial in prospective treatments for anxiety disorders.

## Author Contributions

Conceived and designed the experiments: LX, HS, and XZ. Performed the experiments: LX, HS, XX, and JL. Analyzed the data: LX, PY, and XX. Contributed reagents/materials/analysis tools: LX, HS, PY, XZ. Contributed to grant funding: XZ.

## Conflict of Interest Statement

The authors declare that the research was conducted in the absence of any commercial or financial relationships that could be construed as a potential conflict of interest.
